# A Manual of Midwifery and the Diseases of Women in the Pregnant and Puerperal States, with the Management of Infancy

**Published:** 1847-01-01

**Authors:** 


MANUEL DES ACCOUCHMENTS ET DES MALADIES DES FEMMES GROSSES ET ACCOUCHÉES, CONTENANT LES SOINS A DONNER AUX NOUVEAUX-NES. Par *J. Jacquemier*, Docteur en Medecine de la Faculté de Paris. Avec 63 figures intercalées dans le texte.

A Manual of Midwifery and the Diseases of Women in the Pregnant and Puerperal States, with the Management of Infancy. By *J. Jacquemier*, M.D. With sixty-three figures inserted in the text.

RECHERCHES ET CONSIDÉRATIONS SUR LA CONSTITUTION ET LES FONCTIONS DU COL DE L'UTERUS, dans le but d'éclairer l'Etiologie des Insertions Placentaires sur cette Region, et de conduire a un Choix de moyens propres de combattre les Hemorrhagies qui en sont les Conséquences. Par *C. Negrier*. Paris, 1846.

Researches and Considerations on the Structure and Functions of the Neck of the Womb, with the design of elucidating the Etiology of Insertions of the Placenta in that Part, and to indicate the Proper Treatment of the Hæmorrhages resulting from them. By *C. Negrier*.

THE works before us are the most recent on obstetric medicine from our neighbours in France. M. Jacquemier's Manual is necessarily to a great extent, a compilation, and treats at full length the direct and incidental subjects which belong to midwifery. M. Negrier's is a Monograph on the Anatomy and Physiology of the Neck of the Womb, with the view of illustrating that most important part of practical midwifery, the insertion of the placenta over the os uteri, and the hæmorrhage which results from it.

The Manual is divided into five principal parts. The first includes a description of the Pelvis and the Organs of Generation, considered in their relation to Gestation and Parturition.

The second treats of Fœcundation, Pregnancy and Ovology. The third includes the Diseases of Pregnancy and the Diseases of the Ovum and Fœtus.

The fourth embraces the subject of Practical Midwifery, including Natural Labour and the Varieties of Dystocia.

The fifth is devoted to Puerperal Diseases, and the Management and Diseases of the Infant.

The sixty-three engravings which illustrate the text are immediately copied from Moreau's Atlas, although we believe that M. Jacquemier supplied the preparations from whence Moreau's engravings were taken. We think that the diagrams which M. Chailly has adopted are far better suited for the purpose than the more elaborate drawings which are seen in M. Jacquemier's volume, or in the recent English Midwifery works. They are clear, pointed, and very intelligible, and appear to us, when inserted in the text, to be of great assistance to the student.

The Chapter on the Pelvis, in its normal and morbid states, is very good. M. Jacquemier follows Naëgele in his description of the small and obliquely-ovate pelvis, giving some of his cases at length, and adopting his views. He thinks, with Madame Lachapelle, that the evils resulting from a too large pelvis have been loosely stated, and that the fear of displacement of the womb, abortion, &c. is not warranted by facts.

There is an interesting notice of the secondary effects of congenital or accidental luxations of the femur, and amputation of the leg high up, in deforming the pelvis, for which our author is indebted principally to M. Sedillot.

M. Jacquemier's anatomical descriptions are exact and clear. He details some peculiarities of the cervix of the womb. But M. Negrier enters on this subject at greater length. The latter transcribes in succession various opinions on the disposition of the muscular fibres of this part of the womb, the result of which is in our mind to lead to the impression that they are too intricate to be made out satisfactorily. The general results of Negrier's researches into the arrangement and character of the fibres of the neck of the womb are as follow:

(1.) The tissue of the cervix is of the same muscular nature as the body of the womb.

(2.) The number of fibres is infinitely less in the neck than in the body of the womb—and

(3.) These fibres are not in continuation with those of the body of the womb, excepting only a band posteriorly which descends from the fundus to the lower part of the neck, and even as far as the vaginal cul-de-sac.

(4 & 5.) The number of the circular muscular fibres of the neck, in which two layers are not recognized, as in the body of the womb, diminish towards the inferior aperture, where, according to some anatomists, they collect to form a sphincter.

It is thus that the neck of the womb does not possess, excepting in a very trifling degree, perpendicular fibres which abound in the body of the womb, and hence, so far as muscular fibre is concerned, there is an independence between these two parts.

Besides the difference which is to be noticed in the number and texture of the fibres of the neck of the womb, M. Negrier remarks on the differences in the vascular and nervous supply to these two parts of the same organ. The neck of the womb derives its blood from two large arteries, the uterine, whose primary trunks envelop and penetrate the walls of the cervix, while the body of the womb is supplied from a less considerable source, the spermatic arteries. The cervix possesses spinal nerves principally; the body, nerves from the ganglionic system. And it is impossible, says M. Negrier, but that this disposition must exercise a most important influence in the functions of the organ. Still further to mark the distinction between the cervix and body of the uterus, M. Negrier passes rapidly in review the functions of menstruation, fecundation, parturition, the delivery of the placenta, and the changes which follow labour.

The pains of menstruation, especially those which accompany the expulsion of false membranes, are referred to the difficult passage of the blood along the canal of the cervix. M. Negrier omits to notice the fact that, the cavity of the womb and not the cervix yields the menstrual blood. The peculiar faintness, sometimes amounting to syncope, which is noticed by some women when a fruitful coitus has taken place, results, according to Negrier, from a dilatation of the neck of the womb in its effort to draw in (aspirer) the semen, and the increased sensibility of the part from the venereal orgasm. M. Negrier notices the formation of the decidua from the cavity of the womb, and the closing of the cervix by the plug of mucus. He speaks of the decidua as a pulpy matter, forming after a time a serous sac; and he states that the upper part of the mucous plug, becomes solid, and organized, resembling the pulp of the decidua, and that blood passes into it, which is not held in regular vessels, but imbibed at that part of the plng in contact with the ovum. He noticed this in ova of the fourth to the seventh month. M. Jacquemier's views of the formation of the decidua are similar to those of M. Negrier. He speaks of "its organization as very simple. At first it is not distinct from the fluid secreted by the uterus under the influence of the specific excitement caused by fecundation; a coagulable fluid separating itself into solid parts, which are deposited over the uterus and the ovum in semi-solid layers, offering the most simple form of false membrane; the remainder of the fluid remains in the cavity of the decidua to disappear at a later time." Our authors do not appear to be aware of the investigations of Dr. Sharpey, Mr. Goodsir and others, into the structure of the decidua. We believe that its glandular origin is now indisputably made out. It is in fact nothing more than a swelling of the mucous membrane, the follicles, blood-vessels, and the lining epithelium of the follicles, all growing rapidly. We are somewhat surprized that these most interesting researches should not have been recognized by M. Negrier or M. Jacquemier.

M. Negrier refers the vomiting which attends the early months of pregnancy to a relation subsisting between the stomach and the fundus of the uterus—the latter, in contra-distinction to the cervix, being supplied with organic nerves. It is the dilatation of the fundus during the first period of pregnancy, and the sympathetic effect of this, which occasions the morning and other sickness.

M. Negrier makes a great distinction between the upper and the lower opening of the cervix, the os uteri internum and externum of our anatomists. It is the closure of the upper opening which holds the ovum within the cavity of the womb, and not the parietes of the neck. "The upper opening or sphincter belongs completely, both in its nature and its mode of action, to the body of the uterus—it is *its* mouth, and the inferior aperture or sphincter approaches rather the tissue of the vagina, both in its structure and in some of its functions." There is a difference in the restoration of the neck of the womb after delivery in primiparæ, and in those who have previously borne children; and M. Negrier traces the difference day by day, up to the tenth day after parturition. The result is, that in primiparæ the orifices of the neck close up sooner than the parietes of the cavity of the neck, whilst in multiparæ the lips of the external opening close up more slowly than the walls of the cavity. Amongst all women the walls of the cervix are folded up from above to below, and these perpendicular folds result from the closure of the upper opening of the neck. We shall refer to the practical part of M. Negrier's work by and bye.

The disorders of menstruation are divided by Mons. Jacquemier into two classes—Amenorrhœa and Dysmenorrhœa. In the former, are included the same class of cases as most authors have described under this title, while the latter is greatly extended, and is made to take in Menorrhagia and Vicarious Menstruation. "The line of demarcation between amenorrhœa and dysmenorrhœa is not always well defined. If we except amenorrhœa caused by an imperfect state of the sexual organs, and amenorrhœa which is symptomatic of some other disease, all other known species will more naturally be ranked as dysmenorrhœa."

Our author describes amenorrhœa by obliteration of the vulvo-uterine canal, which, as he justly remarks, is a retention of the menses and not an amenorrhœa—and amenorrhœa the result of a deficiency in the sexual organs, whether in the absence of the uterus or ovaries, or an undeveloped or rudimentary state of these parts.

The Chapter on the amenorrhœa which is secondary to other diseases, contains many of the remarks of M. Brierre de Boismont. Of the physical causes which produce this suppression, the action of cold is the most common. The menses may be stopped by exposing the body when perspiring to the cold air, or by immersing the feet or hands in cold water. But habit diminishes and even destroys this pernicious tendency, and bathing-women are just as regular as other persons. M. Brierre de Boismont says that he has seen cases in which the contact of the surface with cold water caused a very abundant flow.

"The suppression of the sweat of the feet has often been followed by amenorrhœa; it has been seen to follow blows, falls, fatigues, acute diseases which have needed profuse bleedings. The linen which women use during the menstrual times is with some an immediate cause of a diminished flow, and it may even stop it. Moral emotions are the most common causes of sudden suppressions of the menses, although they sometimes occasion a profuse discharge. Both the physical and moral causes act most surely at the approach of the periods, or just when they are on. M. Brierre de Boismont having analyzed, with reference to these causes, 190 instances of suppression, found 68 were due to physical causes, 92 to moral causes, and in 30 the cause was not known."

There are two particular forms of Dysmenorrhœa which are described, the plethoric and the hysteralgic.

Dysmenorrhœa may present itself under several different forms. 1st. With symptoms of active hyperemia, whether local or general. 2nd. With marks of uterine neuralgia, or other nervous symptoms. 3rd. The menses may flow immoderately. 4th. They may shew themselves in other organs besides the uterus.

We think that Monsieur Jacquemier has not done wisely to include the two last disorders of menstruation under the title of Dysmenorrhœa. Already this term is undefined enough, as pain in the performance of a function may represent various and very different pathological states. But it is adding unnecessarily to the confusion to include other disorders of menstruation which are not necessarily attended with local suffering, and have symptoms and morbid actions distinct from them and peculiar to themselves. Our author does not notice that mechanical form of dysmenorrhœa which Dr. Macintosh described, resulting from a congenital narrowing of the os uteri or channel of the cervix, and which yields to the artificial dilatation of the stricture.

M. Jacquemier's description of the plethoric or congestive dysmenorrhœa is imperfect. In it, the flow may be very trifling, notwithstanding the hæmorrhagic movement towards the womb. Sometimes it is entirely suppressed, and resembles the sthenic amenorrhœa of some authors. This form of dysmenorrhœa shews itself principally in the first years of menstruation, and among women who have had profuse periods. In the treatment of it M. Jacquemier says—" if it be not intense, simple expedients, such as rest, diluents, cataplasms to the hypogastric region, &c. may suffice to dissipate it. But if it be more severe, it requires more active treatment, as baths and loss of blood. If the symptoms of congestion appear more particularly in the genital organs, leeches to the upper part of the thighs or vulva may be applied. If on the contrary general symptoms predominate, bleeding from the arm will do more good. M. Roche has often noticed that, in plethoric women, bleeding from the arm just before the period has speedily brought on the flow, which has gone on freely and without pain."

In the hysteralgic form of dysmenorrhœa, our author describes with accuracy the varied symptoms which attend this trying affection. In speaking of the causes which predispose to it, he says that a life of forced celibacy in women of ardent temperament, susceptible of strong attachments, predisposes to it; and those who love a contemplative life, or those devoted to a monastic life, are subject to it. M. Pidoux has noticed a peculiar character in this affection among the nuns. It consists in a disorder of the digestive organs, which is not attended with severe gastralgia, but with a torpor of the stomach, and a sensation of general sinking. This is accompanied with great weakness and mental inaction, against which these women, so strong in their will and courage, are constantly struggling.

We do not think M. Jacquemier very successful in his resources for the treatment of this disease. He talks generally of sedatives, as assafœtida, castor and opium, of diffusible stimuli, warm baths, and purgatives, &c. as means to relieve the pain during the period; and to prevent the return of the hysteralgia, we are directed during the intervals, to improve the general health, and to use antispasmodics, baths, cold affusion, friction, alkaline baths, &c. There is a want of precise direction in his therapeutics, which in a manual is a great defect. Students or young practitioners need something more than this sort of vague generality in the prescription of remedies, and it detracts from the usefulness of a work when accurate directions are lost in undefined plans of treatment.

The Second Book, which is divided into three Chapters, comprises several interesting physiological subjects, and includes one important practical subject—the Diagnosis of Pregnancy. The physiological subjects are on Fecundation, the Anatomical and Functional Changes consequent on Pregnancy, and Embryology. These are generally speaking well written for an elementary treatise, and M. Jacquemier has evidently worked at them himself, which unfortunately is by no means the rule with writers on midwifery. M. Jacquemier does not appear to be acquainted with

Dr. Barry's researches into the anatomical arrangement of the contents of the Graafian vesicle, or the first developmental changes as seen in the ovum of the rabbit, or again of the fact that he has seen a spermatozoon within an ovum, before its escape from the ovary.

This last observation by so accurate and well-practised an observer as Dr. Barry, with the associated fact that, Bischoff had seen spermatozoa on the surface of the ovary in a bitch killed soon after being lined, seems to us to fix the place where the generative elements meet, and that that place is the ovary. We do not participate in M. Jacquemier's prophecy, that the modern theory of menstruation will overturn this observation, because we still doubt whether the periodical casting off of ova during the menstrual periods really takes place.

M. Jacquemier allows that the nerves of the uterns increase in length during gestation, but he seems indisposed to admit beyond this the truth of Dr. Lee's researches. He speaks of Dr. Rendu having drawn attention to a cellulo-fibrous structure which surrounds and protects the nerves of the uterus and vagina, and which presents in some points a ganglionic appearance. We hope soon to see this question set at rest, and we must express our own conviction, in opposition to Dr. Rendu and Mr. Beck, that Dr. Lee has not been deceived by this appearance in the cellular texture, and that what he has described, is the enlarged nervous system of the womb, which, with its vascular and muscular structure, increases with the increase of this organ during pregnancy.

The Third Book contains a full description of the "Modifications produced by Pregnancy which take on Morbid Characters," or, in other words, the Diseases of Pregnancy. These consist of Ptyalism, Odontalgia, Sympathethic Affections of the Stomach, Constipation, Diarrhœa, Plethora, Palpitation, Syncope, Œdema of the Lower Extremities, Hæmorrhoids, Fever of Pregnant Women, Sympathetic Affections of the Nervous Centres, Diseases of the Mammæ, Relaxation of the Symphyses of the Pelvis, Distension of the Abdominal Wall, Pains in the Loins, Affections of the Urinary Apparatus, Pruritus of the Vulva, Leucorrhœa of Pregnant Women, Uterine Hydrorrhœa, Pains in the Uterus.

This Book contains also a section on Extra-Uterine Gestation, another on the Displacements of the Pregnant Womb, a third on the Diseases of the Ovum, a fourth on the Diseases of the Fœtus in Utero, a fifth on Abortion and Uterine Hæmorrhage during the First Six Months of Pregnancy, and lastly, a Section on the Influence which Pregnancy has on Intercurrent Diseases, and their reciprocal influence on it. In this Section also the Hygiene of pregnant women is considered.

M. Jacquemier speaks favourably of bleeding as among the more active remedies for the stomach affections of pregnant women, and he regards the danger which is supposed to attend local bleedings during gestation as greatly exaggerated. We think, with our author, that small bleedings during pregnancy are frequently well borne, and relieve many of the painful affections of this state. It is not an expedient adopted sufficiently often in this country, and we think it merits more attention.

Among the diseases of pregnancy, we notice a paragraph on Uterine Hydrorrhœa, or the *fausses eaux* of other writers. The seat of this serous discharge is not yet determined. The fluid resembles the liq. amnii in color and consistency. It is generally colourless, sometimes of a light yellow, sometimes slightly tinged with blood; its odour recals that of the fœtal appendages. Its quantity is variable, generally moderate, but sometimes very considerable. These serous discharges may occur only once, or at different times during one period of pregnancy, and rarely at any other period than the last. There is no appreciable morbid state which either precedes or accompanies them. Sometimes there is a plethoric and at others a feeble state of health. Occasionally they come on during the night, at others in the day time, and sometimes after any movement or emotion. When they appear during labour, they are brought on by the contractions of the womb, and are taken usually for the liq. amnii. In general these discharges are perfectly harmless, neither interfering with gestation or the health of the fœtus. On examining the membranes after delivery, there is no physical lesion which can prove that they have given way. M. Jacquemier does not believe that the fluid is collected between the amnion and the chorion, nor that it is the hydroperionic fluid in excess, and the absence of hydatids on examination of the fœtal appendages forbids the assumption that they have caused it. M. Naëgele thinks that these discharges do not come from within the ovum, but that there is a partial inflammation of the inner surface of the womb, and that a serous fluid is thrown out between this surface and the decidua, and that the uterus contracting on it forces the fluid gradually towards the neck of the uterus, detaching the decidua in its course, and at length it escapes. M. Jacquemier does not feel constrained to admit an inflammatory process, of which there is no evidence, and he speaks of two cases in the Clinique of M. Dubois, in which the uterus evidently furnished a serous discharge after delivery in place of the lochia. In both these cases the women had had serous discharges during pregnancy. M. Jacquemier is not aware that, many years ago, Dr. Ashwell wrote a paper in the Medical Gazette, entitled Aqueous Discharge after Parturition, in which a clear pellucid serum was described as the substitute for the lochia. We do not remember in Dr. Ashwell's cases the occurrence of uterine hydrorrhœa during pregnancy; and, in a case which we are ourselves cognizant of, a lady has had these aqueous lochia after three successive pregnancies, but no such discharge during gestation. M. Jacquemier very properly observes that, the doubt as to the source of the fluid is of little moment comparatively, as hydrorrhœa is not an important or dangerous disease.

The Chapter on Abortion and Early Hæmorrhages is full and practical, but it does not contain any views of a peculiar or novel kind.

The concluding Section of this Book treats on a subject of much interest, which is scarcely mentioned in the various elementary treatises on midwifery. "The influence of pregnancy," says M. Jacquemier, "on diseases which are strangers to it, and these again on pregnancy, has not hitherto been studied with the care it deserves." "Is the economy," he asks, "during pregnancy more protected against morbid actions, or is it more apt to receive them, or is there not in this respect any appreciable difference?" M. Jacquemier states his belief that pregnancy, speaking generally, is a protection against disease, but it is no certain security against the influence of any one disease. In epidemics, such as the cholera in 1837, most of the pregnant women in the Maternity were attacked; and the history of this disease, and most other epidemics, proves that pregnant women are not more exempt than others. "Small-pox is said to be almost always fatal during pregnancy, and scarlet-fever and measles, although less severe, ought to awaken the greatest solicitude when they are intense enough to bring on abortion or premature labor." We are not disposed to think that variola or scarlet-fever during pregnancy is often met with. We have often been struck with the immunity of pregnant women against the infection which may be spreading around them, and no less so with the power which pregnancy has of postponing the development of the exanthemata until after delivery.

M. Jacquemier says that acute bronchitis attacks pregnant women very frequently. In the Maternity he noticed that the women who were enceinte were much more frequently the subjects of acute bronchitis than the éleves sage-femmes, and he is disposed to think that pregnancy predisposes to acute bronchitis. It does not, however, dispose to other acute diseases, for, although our author has searched both ancient and modern records, he has found but very few cases in which pregnant women were attacked with pneumonia, pleurisy, articular rheumatism, or typhoid fever.

M. Grisolle's observations on fifteen women attacked with pneumonia during their pregnancy are quoted. Of these, ten had not reached the sixth month—whilst five approached the seventh, eighth, and ninth month. Of the ten in whom pregnancy had not arrived at the sixth month, four aborted at the fourth, fifth, sixth, and ninth days from the commencement of the disease. In three, abortion was followed by a severe affection of the side of the chest, and death occurred in three or four days afterwards. One alone, in which the pneumonia had not gained ground, recovered well. The six women who did not abort died from the pneumonia. Of the five women who had arrived at a later period of gestation—two were attacked at the seventh month of pregnancy, and a premature labour at the twelfth and fifteenth day took place, the expulsion of the fœtus preceding only by two days this disastrous termination. Of the remaining three, two gave birth to living children at the seventh and eighth day of the disease, and the other died on the fifth day, undelivered. The Cæsarian section was performed, but the infant was dead.

Mons. Grisolle's other observations on acute febrile diseases, such as enteritis, acute bronchitis, pleurisy, erysipelas of the face and head—indicate a favourable termination without interrupting the course of pregnancy, with the single exception of a typhoid fever which ended fatally.

M. Jacquemier noted three cases of acute articular rheumatism in women arrived at an advanced period of fœtation. The disease was treated energetically on the antiphlogistic plan, but without removing it. These three women were confined at term of living children; in two of them the symptoms speedily disappeared after delivery, but, in the third, the rheumatism was fixed in the knee, and remained there for three months after parturition. When acute diseases are not intense enough to produee abortion the patients are not in a worse condition for treatment than women who are not pregnant, but when abortion takes place before the disease is cured, it is generally fatal. "The saying of Hippocrates, that acute diseases in pregnant women are mortal, is full of truth when applied to their producing abortion." We suppose our author is alluding here to the fact, that before death occurs in acute diseases, the uterus usually casts off the ovum—which has been particularly noticed by Dr. Montgomery and others.

Chronic maladies are said by M. Jacquemier to be rarely developed during pregnancy. In females affected with pulmonary phthisis, which has not yet reached the hectic stage, pregnancy goes on well to the full period, and what is more remarkable, they give birth to vigorous and well-developed children. On the other hand, the progress of phthisis is apparently often modified. In some cases, phthisical patients gain flesh, their general health undoubtedly improves, although tuberculization and disorganisation may be going on. Sometimes, however, but not constantly, there is a real arrest of the disease; most commonly, it follows its usual course, occasionally becoming more rapid, especially when it is much advanced. Patients die frequently a few weeks after delivery at full term, and sometimes they sink before delivery.

The Fourth Book is devoted to Midwifery. We find M. Jacquemier treating this part of his subject with much care and practical knowledge. His book is a sound midwifery book. Our limits will not permit us to follow him in the various divisions of his subject. He has extended the term natural labor, so as to include pelvic and face presentations, and the natural labor of twins. The phenomena of natural labour, its mechanism, involving the mechanism of cranial, face, and pelvic presentations, and the management of labor are clearly, perspicuously, and scientifically described.

The causes of dystocia are ranged under four sections. In the first section are the cases in which labor is impeded by an error in the expulsive force, and those in which, the presentation and the other conditions of labor being natural, the power of expulsion alone is enfeebled. The majority of the labors which come within this class are more painful, laborious and dangerous than natural labors, but are frequently terminated spontaneously, requiring only a longer time and general remedies. The cases of dystocia which have reference to the fœtus, such as prolapsus or shortening of the cord—hydrocephalus, hydrothorax, &c., form the second section. The third comprehends labors rendered difficult from mechanical obstacles formed by the parts of the mother. In this, we have labors with deformed and contracted pelves, with the presence of tumours both without and within the vulvo-uterine canal, with contractions or malformations of the soft parts. It comprehends, too, labors rendered faulty by the various displacements of the womb, and labours during attacks of acute diseases. The fourth section is on accidental dystocia, such as convulsions, hæmorrhages, ruptures of the uterus and vagina, &c.

The remainder of the Book is taken up with Operative Midwifery, including the induction of premature labor, and the difficulties which may occur in the delivery of the placenta.

M. Jacquemier admits the reputed power of the ergot of rye in increasing uterine action, which he says is almost constant. He fences the use of this medicine by several acknowledged precautions, although, contrary to the opinion of most authorities, he thinks it may be used before the os uteri is completely open, if the membranes are unruptured. In moderate doses the ergot is harmless to the mother and the child, but if, after the liq. amnii has passed off, the child is subject to the influence of the contractions produced by ergot, the effect on its circulation is more likely to be fatal than under natural contractions. "It has been supposed," says M. Jacquemier, "that the infant may be attacked with a sort of intoxication, but this is by no means proved, and before it is possible, we must suppose that the ergot has been administered during a long time and in large doses." It is evident that our author is not acquainted with the researches of Dr. Beatty and Mr. Hardy, or we think he would be more willing to regard the ergot as a poison to the child. The peculiar form of convulsion seen in some children who have been subject, during their birth, to its influence through the mother for more than two hours, is too characteristic of the poison of ergot to be ascribed to the mechanical pressure from increased or unintermitting uterine action. We think that Dr. Beatty's cases fully bear this out, and the concurrence of both these authors, in the depressing influence of the ergot on the mother's pulse, is an interesting fact which M. Jacquemier does not notice.

The spontaneous return of the head under a shoulder-presentation is noticed by M. Jacquemier under the title of cephalic version. He quotes an illustrative case from M. Velpeau, in which this author traced the gradual removal of the head from the right iliac region to the brim of the pelvis, which it entered, and the child was born in the right occipito-cotyloid position.

In the treatment of trunk-presentations, our author enters very fully into the question of the ancient operation of cephalic version. He quotes numerous authorities in favour of it—Mauriceau, Smellie, La Motte, Osiander, Flamant, and others. M. D'Outrepont reports five cases in which cephalic version completely succeeded; but in three of them by external manipulation, probably before the liq. amnii had passed off. On this mode of cephalic version, viz. before the membranes are ruptured, M. Jacquemier quotes 15 cases from Busch, in four only of which the version was performed *after* the rupture of the membranes. All the children were born alive. In the midwifery statistics in the kingdom of Wurtemberg, from July 1821 to July 1825, published by M. Rieche, sixteen examples are recorded of cephalic version after the opening of the membranes. One child only was lost, and the several mothers did well. To the cases already published in 1827, M. Ritgen adds 13 others. Siebold, Carus, Joerg and Burns have spoken favourably of this practice. M. Velpeau is the first author in Paris who has reported in favor of cephalic version, although he has not practised it himself. Our author, however, like M. Dubois, restricts this practice, admitting its possibility, to a limited number of cases. We abridge his conclusions on this point.

1. It cannot be attempted if the os uteri is not dilated, or dilatable enough to admit the hand, at the moment of the rupture of the membranes.
2. When some time has elapsed since the escape of the liquor amnii, and the uterus is strongly contracted on the child.
3. When the child is dead.
4. In premature labors.
5. When the arm escapes.
6. When the cord prolapses, unless the child has not yet suffered from its compression.
7. Any complication, the failure of the forces of labour, &c. ought to contra-indicate this version, as its advantages would be more than balanced by the probable use of the forceps to effect delivery.

For further information on this subject we must refer our readers to M. Jacquemier's excellent Chapter on Trunk-presentations.

In the accidental form of dystocia, M. Jacquemier considers uterine hæmorrhage in the latter months of pregnancy. This subject includes both accidental and unavoidable hæmorrhage, and M. Negrier also treats of them in his work.

M. Negrier endeavours to explain the cause of a placental-presentation. He reverts to his researches on the human ovaries, in which he claims the merit of having started the recent theory of menstruation, and he states "that he has there proved that every menstrual epoch is the consequence of the development, separation, and escape of an ovum from a Graafian vesicle. This ovum, carrying a living but *unimpregnated* germ, is seized by the tube, and almost always conducted by it to the cavity of the uterus. This ovum is carried on towards the inferior angle of the cavity by the menstrual blood, if the blood is still flowing, or by the whitish mucus which follows the menstrual exudation." M. Negrier thinks that the ovum may be fecundated at any point between the ovary and the outlet of the womb, wherever the semen may meet it; and that in a placenta-presentation the ovum must have arrived at the internal orifice of the womb, where it becomes fixed.

In a normal fecundation the decidua is forming before the ovum gets into the uterus, and it is the means by which the ovum is confined to the upper part of the womb ; but, in abnormal presentations, the ovum not yet impregnated, is conducted into the uterine cavity before the arrival of the semen or the formation of the decidua.

A partial placenta-presentation results from the ovum having arrived near the inferior aperture of the cavity of the womb before it is fecundated, and a complete placenta-presentation implies that the ovum should have got still nearer the lower angle. When it is impregnated at this place, the chorion throws out its villi every where around the superior aperture, and the placenta is fixed, centre pour centre—over the os uteri internum.

M. Negrier explains the well-known fact that, in some placenta-presentations, hæmorrhage occurs at the end of the 5th month, and, in others, that it is postponed to a later period, by supposing that, in the latter cases, some of the radicles of the placenta, towards the middle of gestation, descend into the upper opening and become fixed in the walls of the cervix itself, and this part of the placenta is not, therefore, stretched out and disrupted, as the cervix is taken up during the latter months of gestation. The early hæmorrhages occur from the placenta lying over the os internum, and as the placenta does not yield to the development of the neck, its structure is lacerated, and hæmorrhage ensues. It is then the central insertions which are attended with early and copious bleedings.

M. Negrier illustrates the subject of uterine hæmorrhage by narrating and commenting on two sets of cases; first, those connected with a normal insertion of the placenta at or near the fundus of the uterus, the bleeding in these cases coming from the walls of the cavity of the womb; and second, those bleedings coming from the inferior part of the womb, and from the *inner surface of the cavity of the neck,* on which parts the placenta has been engrafted.

M. Jacquemier designates the first class of bleedings (the accidental hæmorrhage of English authors) as an utero-placental hæmorrhage, of which there are two varieties, the first internal or latent, the latter external or apparent; whilst M. Negrier applies the term cervico-placental to the unavoidable forms of bleeding.

With reference to the first class of bleedings, M. Negrier relates twelve cases, all of which are described in a clear and direct way; and the reflections which accompany them are generally judicious and appropriate. The first six cases are examples of hæmorrhage either before or during parturition. The seventh is a case of latent bleeding after delivery, with a second bleeding twelve days after labor, coming on when the child was sucking. The eighth also is a case of hæmorrhage a month after delivery, brought on by the act of sucking. The two succeeding cases are examples of adherent placenta with hæmorrhage, and the twelfth is a natural labor, in which the perineum was lacerated, hæmorrhage followed delivery, then metritis, which was cured. On the 18th day after delivery the patient was walking about. On the 29th day the patient walked about alone and without assistance, and on the same day, having eaten more freely perhaps than she should have done, she experienced a strong emotion of joy, and at the same moment spasms, abdominal swelling, particularly at the epigastrium, constant vomiting, considerable fever, came on—and the following day she died. No inspection was permitted.

It will be seen that these cases are in many respects very dissimilar, but they agree in the one thing which the author had in view, namely, that the hæmorrhage in all of them came from the body of the womb. The remarks on the first case appear to us to contain the gist of the author's design in relating them, which is to draw a distinction between the bleedings from the upper and the lower part or the neck of the womb. After relating the case M. Negrier says—"In this case the blood flowed regularly—without intermittent pains—without increase up to the last; it was blackish. These signs suffice to shew that the bleeding came from the fundus of the womb from a detachment of the placenta. It would have been otherwise if the placenta had been near the upper opening—the flow would have been more red—it would have been irregular, and would not suddenly have ceased after the expulsion of the ovum." In the remarks on other cases, M. Negrier notices also the effect of contraction in diminishing the accidental, and increasing the unavoidable hæmorrhages, and he insists also on the increased danger which attends the latter cases.

There are eight cases related by M. Negrier of hæmorrhage, caused by detachment of the placenta inserted into the neck of the uterus. The deaths and recoveries in these cases are equally divided, there being four of each. The abbreviated account of each case, as shewn in the heading of them, is as follows:—

*Case* 1.—Insertion of the placenta over the neck of the uterus—placentar adhesions—plugging—recovery—child alive—spontaneous delivery.

*Case* 2.—Insertion of the placenta over the neck of the uterus—extensive placentar adhesions—copious bleedings—plug—recovery—spontaneous delivery.

*Case* 3.—Very large insertion of the placenta over the neck of the uterus—frequent bleedings during the last three months of gestation—insufficient labor-pains. Forceps applied at the brim. Death of mother and child.

*Case* 4.—Large insertion of placenta over the neck of the uterus—hæmorrhage—version—compression of the aorta. Death of mother—child alive.

*Case* 5.—Large insertion of the placenta over the neck of the uterus—copious hæmorrhage—ergot—forceps—compression of aorta. Death of mother and child.

*Case* 6.—Very large insertion of the placenta over the uterine neck—hæmorrhages—ergot—version—compression of the aorta. Death of mother—child alive.

*Case* 7.—Large insertion of the placenta over the neck of the uterus—frequent hæmorrhages—plug—ergot—version—perpendicular pressure over the body of the womb. Recovery of mother—child alive.

*Case* 8.—Large insertion of the placenta over the neck of the uterus—vaginal plug—version—perpendicular pressure of the uterus. Recovery of mother—child restored to life.

M. Negrier devotes a chapter to the prognosis of hæmorrhage from placentar presentations. He quotes several opinions on the extreme danger of this accident, but he prefers the following laconic expression of M. Naëgele. "When these hæmorrhages are abandoned to themselves, or relieved too late, they almost always end in death."

M. Negrier says, too, that "the cervico-uterine bleedings are almost the only ones which kill. Doubtless bleedings from the walls of the body of the uterus may be fatal, but such cases are exceptions."

Although the early bleedings at the sixth and seventh month indicate a large insertion of the placenta at the cervix, still M. Negrier does not regard these cases as so dangerous as those where, towards the close of gestation, a sudden and large detachment of the placenta takes place, especially if it occur in a multipara. He considers the most unfavourable case possible to be as follows:—"The placenta shall be very largely grafted on the walls of the cervix, but, notwithstanding this large implantation, the cervix should have increased till the normal term of gestation. It should not be a first pregnancy, and labor should have set in energetically and suddenly, by a very large separation of the placenta. In such circumstances, the hæmorrhage is so sudden and copious, that it almost always causes the death of the patient, whatever may have been the skill and promptitude of the treatment."

The third and concluding part of M. Negrier's treatise is occupied in the treatment of the hæmorrhages which follow a detachment of the placenta. This he divides into three parts. 1. The treatment of hæmorrhage during gestation. 2. During labour, including the unavoidable bleedings from placenta-presentations. And 3. Post-partum hæmorrhage.

It is almost a constant practice, says M. Negrier, to bleed women who are attacked with hæmorrhage before the seventh month of pregnancy. Our author, however, does not fall in with this axiom. His views on this subject are thus summarily stated.

Bleeding from the arm is a uselessly wasteful means to combat hæmorrhages from detachment of the placenta.

Bleeding is useless for the preservation of the fœtus whenever a third of the placenta is detached ; and M. Negrier thinks that this is indicated with sufficient exactness when 60 grammes* of blood flow in the course of an hour, and, excepting in special cases, such a loss will prevent gestation going on, in spite of bleeding.

Bleeding is particularly hurtful in placenta-presentations. An *exact* plugging of the vagina is the only effective means to suspend copious bleedings during gestation. The plug ought to be covered with a greasy and tenacious matter, and it ought not to be impregnated with vinegar.

In the second class of cases, where hæmorrhage comes on during labour, M. Negrier insists on the great importance of determining without delay whether the placenta presents or not. If it does not, and the os uteri is closed, the plug ought to be used to arrest the bleeding. When labor has set in actively, and the os uteri is supple, then the membranes ought to be perforated. This plan is contra-indicated if the child presents transversely, or if the pelvis is contracted. If the hæmorrhage continues after the rupture of the membranes, the plug ought again to be used, for the double purpose of arresting the bleeding and exciting the uterus to contract. The ergot of rye is now too very useful, excepting only when exhaustion from loss of blood is present, when it will not act. The uterus ought to be followed as the child is expelled, and its contraction secured by friction or circular compression of the belly. Stimulants are more needed at this time than opiates. The forceps ought only to be used when the head is low down in the pelvis, and well placed.

In the treatment of placenta-presentations, if the detached portion is large, and the loss of blood is great, every effort ought to be made to empty the womb, and version is the most prompt and efficacious means for this purpose. If the os is unopened it ought to be forcibly dilated (forcé)—and in women who have had children it rarely offers any dangerous resistance. To perforate the ovum with the hope of lessening the bleeding, is a useless and absurd practice. When the orifice of the womb is completely blocked up by the placenta, we ought to search for the side on which it is stretched out the least, and detach the placenta from this part. If the placenta is very *largely* attached, and is adherent and thick to the touch—the centre pour centre insertion—M. Negrier would, without hesitation, perforate it, in order to turn the child. Fœtal hæmorrhage from the tearing of the placenta is rarely dangerous, if the extraction of the fœtus is quickly executed. The tearing away of a loose portion of the placenta to stay hæmorrhage can only be useful when the uterine neck preserves the form and consistence of a canal.

M. Negrier ends this chapter by observing "that the complete separation of the placenta before the extràction of the fœtus, and its expulsion from the uterus before the escape of the child, do not bring any modification to anti-hæmorrhagic means—they are complications which render the speedy emptying of the womb more imperious."

This is the general outline of M. Negrier's views on the cause, symptoms, diagnosis, prognosis, and treatment of placenta-presentations. We cannot but think that he has added very little to what was well known

* A gramme is equal to 15·438 grains Troy weight.

upon the subject. We need scarcely say that it is one of special present interest—an interest founded on the views of Dr. Radford and Professor Simpson. It appears from the last paragraph which we have quoted, as well as from a long foot-note, that M. Negrier is not ignorant of the recent investigations on this subject. The note which we refer to is a running commentary on Dr. Radford's series of propositions on the class of cases in which the artificial separation of the placenta and the use of galvanism is applicable. They were published in the fourth number of the second volume of the Provincial Medical and Surgical Journal, and were copied into the Journal de Chirurgie, which is the source of M. Negrier's information upon them. It is beside our purpose to enter critically into the views of Dr. Radford and Dr. Simpson; but, in order to comprehend M. Negrier's comment, we copy at length Dr. Radford's propositions.

First. As neither delivery nor detaching the placenta ought ever to be attempted until the cervix and os uteri will safely allow the introduction of the hand, rest, the application of cold, but above all the use of the plug, must never be omitted in cases where they are respectively required.

Secondly. If there are unequivocal signs of the child's death, the placenta is to be completely detached and the membranes are to be ruptured. The case is then to be left to the natural efforts, provided there be sufficient uterine energy, if otherwise, the ordinary means are to be used, and in addition galvanism.

Thirdly. When a narrow pelvis exists in connexion with placenta previa, the practice is to detach the placenta and to remove it, then to perforate the head as soon as the condition of the parts allow, and to extract it by means of the crotchet.

Fourthly. When the os uteri is partially dilated and dilatable, so as to allow the easy introduction of the hand, when the membranes are ruptured and strong uterine contraction exists, the practice is to detach the placenta completely.

Fifthly. In all cases of exhaustion, as already referred to in my paper,* the practice is to draw off the liq. amnii by perforating the placenta, as there recommended, then to detach completely this organ and apply galvanism.

Sixthly. In all cases of partial presentation of the placenta, the artificial rupture of the membranes will generally be found sufficient to arrest the hæmorrhage, but if that should prove ineffectual then we must apply galvanism.

Dr. Simpson has also shortly indicated a class of cases in which the artificial detachment of the placenta may advantageously be had recourse to. " I believe it will be found," he says, " the proper line of practice in severe cases of unavoidable hæmorrhage complicated with an os uteri so insufficiently dilated and undilatable as not to allow with safety of turning; in most primipara; in many of the cases in which placental presentations are (as very often happens) connected with premature labor and imperfect development of the cervix and os uteri; in labors supervening earlier than

* Lecture on Galvanism, applied to the Treatment of Uterine Hæmorrhage. Provincial Medical and Surgical Journal, Dec. 24th, 1844.

the seventh month; when the uterus is too contracted to allow of turning; when the pelvis or passages of the mother are organically contracted; in cases of such extreme exhaustion of the mother as forbid immediate turning or forced delivery; when the child is dead, and when it is premature and not viable."

In these views of the above-quoted authorities, M. Negrier does not participate. He has intentionally, he says, omitted to speak of galvanism, because it has not the sanction of French accoucheurs. Even admitting the facts of Dr. Radford as to the powerful influence of galvanism, he doubts its power in contracting the walls of the neck of the uterus, because this part does not, in his opinion, enjoy the sort of contraction which the walls of the body of the womb do. "Dr. Radford's (erroneously written Bradford) first and sixth propositions expressed only admitted facts. The third proposition is irrational. It is at least useless in a case of pelvic deformity requiring craniotomy previously to detach and extract the placenta."

According to M. Negrier's anatomical and physiological views, the practice which is advocated in the fourth proposition is dangerous and useless.

With reference to the fifth proposition, M. Negrier speaks of the proposed operation as the more rational, because the placenta itself, which under the influence of induced labor would necessarily be detached, would offer but little impediment to the passage of the child.

He thinks that galvanism can only be used in maternity charities, as it would take at least one hour to get the apparatus in order for use, supposing a practitioner possessed it, and this delay in severe cases is not often allowed to the accoucheur. What too would country practitioners do unless they carried it always with them? M. Negrier makes no further commentary on Dr. Simpson's papers. M. Jacquemier has not noticed the subject.

It appears to us that M. Negrier does not in the least appreciate the important fact, that hæmorrhage is known to cease when the placenta is completely separated from the surface of the womb. Dr. Simpson has undeniably proved this from the 141 cases which he has collected, in which the placenta was expelled or extracted before the child. We apprehend that this standing fact does not admit of controversy. The reputed fatality of the operation of version in placenta-presentations, as gathered from statistics, may be, and we think is, overrated. Dr. Simpson may or may not be right as to the source of hæmorrhage in these cases; the placenta may not be so readily detached in difficult cases as Dr. Simpson supposes, but nothing of this kind affects the fact, that in a case of unavoidable hæmorrhage, where a woman's life is in danger from bleeding, and the womb cannot be emptied by turning, that the bleeding may be stopped by separating and removing the placenta. For our own part we are disposed to accept this fact, and the practice founded on it, as a most important aid in these most trying and anxious cases. It is not designed to supersede the practice of turning, but it is designed to come into operation where this great resource fails us. Since Dr. Radford's and Dr. Simpson's papers several cases have been recorded, bearing out the practical views of these accoucheurs, and we have no doubt but that they will eventually be established as rules of practice in certain cases of unavoidable hæmorrhage.

As to the value of galvanism in these and other cases, we still need a large collection of facts, before its just value as a remedial agent can be established. The most recent investigations on it, as applied to midwifery, do not promise so much for it as might have been expected. But we hope Dr. Radford will again publish on this subject.

We shall not follow M. Negrier in the treatment of post-partum hæmorrhage. He advocates the use of the plug in all such cases, with external pressure from above. The compression of the aorta, he says, does not entirely suspend a bleeding coming from the cervix. It is a dangerous expedient, as it occasions loss of most precious time, and severely affects the functions of circulation and respiration.

M. Jacquemier's Chapters on Version, and Operative Midwifery, are practical and well written. He is what may be called an advocate for the use of the long forceps, but he counsels a careful and prudent attention to the class of cases in which they are serviceable, and to the manner of employing them. He has constructed a comparative table of the results of forceps and craniotomy cases in public establishments at different parts of the world, which exemplifies the evident difference in the proportion of cases in which they are used. Dr. F. Ramsbotham's statistics of the Royal Maternity Charity shew a proportion of 1 forceps case to 785 deliveries, whilst the cases of Siebold of Berlin, and Carus of Dresden, are respectively 1 in 7 and 1 in 14! M. Jacquemier regards the vectis as a very inferior tractor to the forceps. He exposes the abuse and fallacy of its employment in Holland, by stating that Bruyn pretends to have dislodged 800 heads in the space of 42 years. We quite agree with our author in thinking that those who pretend to use this instrument so successfully have usually employed it in cases where Nature was equal to the task, if time and patience were only allowed. In France, the vectis is principally used to redress the head.

In the operations for Embryotomy, M. Jacquemier advocates the use of Baudelocque's cephalotribe, of which there is a drawing. Among the obstetric operations, M. Jacquemier has included the induction of premature labor. The subject is very well discussed, and this section is a very valuable one. He considers it of the greatest importance to distinguish carefully between premature labor and the induction of abortion, and he thinks it is by having confounded the two that there has been so much repugnance shewn in adopting the expedient of artificial delivery at the seventh month on the Continent. Our author gives a concise history of the introduction of this operation. In 1756, according to Denman, several medical men in consultation unanimously declared this practice to be useful and moral. In a short time, Macaulay tried it, and Kelly practised it three times on the same woman. In a short time it passed into general practice. In Germany, although Wenzel practised it in 1804 and 1808, and Krann in 1819, it was not generally adopted until the facts published in England were known, and Reisenger's work in 1820 was circulated. From Germany it soon spread into Holland and Italy. In France it was resisted for a long time, and it is only recently, from 1832 to 1835, that the works of Dezeimeris, Velpeau, Dubois, &c. have triumphed over the prejudices with which it was regarded. M. Jacquemier indicates the class of cases in which it ought to be used, but he has not mentioned Dr. Ashwell's name in connection with the induction of labor in cases where the uterus is affected with hard and fibrous growths.

The direct or mechanical means to bring on labor are described as—1, Friction over the fundus and neck of the womb. 2. The detachment of the lower segment of the membranes by the finger. 3. Plugging the vagina. 4. Puncturing the membranes. 5. The introduction of some foreign body into the neck of the uterus.

In England the preference is, we believe, generally given to the artificial rupture of the membranes by puncturing either low down, or by conducting the instrument higher up between the uterus and the membranes, to let the liq. amnii escape from this part of the ovum. In France, however, the dilatation of the neck of the uterus by a sponge-tent is the plan most usually adopted. Frictions, the detachment of the membranes, plugging the vagina, &c. are, says M. Jacquemier, but imperfect varieties of this method. MM. Busch and Mende have proposed to dilate the neck by means of three-bladed instruments, which can stretch out after being introduced. M. Dubois uses a speculum to aid the insertion of the tent, which may be retained in the neck by plugging the vagina with sponge. The patient may walk about, and the sponge swells out as it gets moist, and so keeps up a more active dilation.

The subjects of Symphysiotomy and the Cæsarian Section are discussed at length, and the concluding Book, which includes puerperal diseases, and the accidents to the infant from labor, and the management of the infant are practical, and well deserve a careful perusal.

The impression which an examination of M. Jacquemier's Manual has left on our minds is most favourable. It is undoubtedly the best book of the kind now extant. It is a full book—but it is not prolix. It is a scientific book—but it is sensibly written and very practical. We feel persuaded that it will be extensively circulated among French students—and it would give us much pleasure if this notice of it were to attract the English student of midwifery to study it. We confess, on looking at the Preface, we were a little afraid that our author would disappoint us. There are scraps of foolish and peculiarly French vanity, which turn out, however, to be very innocent. In speaking, for instance, of the progress of obstetric medicine during the last and present century, he says that "France having been in this branch of science, as in all others, at the head of the movement, and having filled the world with the noise of her works, cannot consent to be placed behind England or Germany in obstetric science—she must preserve her authority in the midst of these active and fertile nations." We of course are only amused at this little specimen of sectional patriotism, and we are happy to assure our readers that they will not meet with any such vapid nonsense in the work itself.

